# Technological Application of Maltodextrins According to the Degree of Polymerization

**DOI:** 10.3390/molecules201219746

**Published:** 2015-11-27

**Authors:** Zenaida Saavedra-Leos, César Leyva-Porras, Sandra B. Araujo-Díaz, Alberto Toxqui-Terán, Anahí J. Borrás-Enríquez

**Affiliations:** 1 Academic Coordination, Altiplano Region, Autonomous University of San Luis Potosi, Road Cedral km. 5+600, 78700 Matehuala, San Luis Potosi, Mexico; 2 Advanced Materials Research Center (CIMAV), Alianza Norte 202, Research and Technological Innovation Park (PIIT), 66600 Apodaca, Nuevo Leon, Mexico; cesar.leyva@cimav.edu.mx (C.L.-P.); alberto.toxqui@cimav.edu.mx (A.T.-T.); 3 Doctorate Institutional in Engineering and Materials Science (DICIM), Sierra Leona 530, Lomas, 2nd. Section, 78210 San Luis Potosi, San Luis Potosi, Mexico; sandra_berad@hotmail.com; 4 Faculty of Chemistry Sciences, Autonomous University of San Luis Potosi, Manuel Nava 6, 78290 San Luis Potosi, San Luis Potosi, Mexico; anajo_borras@hotmail.com

**Keywords:** maltodextrins, degree of polymerization, water activity, glass transition temperature, overall appearance, technological application

## Abstract

Maltodextrin (MX) is an ingredient in high demand in the food industry, mainly for its useful physical properties which depend on the dextrose equivalent (DE). The DE has however been shown to be an inaccurate parameter for predicting the performance of the MXs in technological applications, hence commercial MXs were characterized by mass spectrometry (MS) to determine their molecular weight distribution (MWD) and degree of polymerization (DP). Samples were subjected to different water activities (a_w_). Water adsorption was similar at low a_w_, but radically increased with the DP at higher a_w_. The decomposition temperature (T_d_) showed some variations attributed to the thermal hydrolysis induced by the large amount of adsorbed water and the supplied heat. The glass transition temperature (T_g_) linearly decreased with both, a_w_ and DP. The microstructural analysis by X-ray diffraction showed that MXs did not crystallize with the adsorption of water, preserving their amorphous structure. The optical micrographs showed radical changes in the overall appearance of the MXs, indicating a transition from a glassy to a rubbery state. Based on these characterizations, different technological applications for the MXs were suggested.

## 1. Introduction

Maltodextrin (MX) is a polysaccharide produced from the acidic or enzymatic hydrolysis of starch which has a nutritional contribution of only 4 calories per gram. It is considered a polymer of d-glucose chains linked by glycosidic α-(1-4) and α-(1-6) bonds, and is formed by linear (amylase) and branched (amylopectin) carbohydrates with different equivalents of dextrose (DE) [1]. Traditionally, the way to chemically characterize commercial polysaccharides containing reducing sugars is by the DE, which is an indirect measure of the free glucose functional groups (aldehydes) responsible for granting the reducing power [2]. Maltodextrin is the name given to those hydrolysates with DE values in the 3–20 range, while those with values higher than 20 are known as glucose syrups. In this sense, the physicochemical and functional properties of MXs depend on the DE value *i.e.*, powder may behave as a highly hygroscopic powder or as a concentrated liquid solutions [3]. The DE is inversely related to the number-average molecular weight (M_n_) and the degree of polymerization (DP), both commonly employed for describing the size distribution of the polysaccharide chains in the carbohydrate polymer. The importance of the M_n_ is related with the prediction of the colligative properties such as boiling and freezing temperatures, while the DP is related with the polymer chain mobility, where larger chains have limited movement [4]. In the food industry MXs have been widely employed, providing different benefits such as the improvement of texture, reducing floury taste, as sweetness modifiers, controlling non-enzymatic browning, decreasing the freezing point in mixtures, as carriers and ingredients [3,5]. Likewise, because of their high solubility in water, MXs are widely employed in the encapsulation industry, mainly as additives in the drying processes [6,7]. The most important benefit of the addition of MX is increasing the T_g_ of the food product and the subsequent preservation of the physicochemical properties.

Among the drying methods used for producing powdered foods (e.g., hot air fluidized bed, freeze drying, flash drying, *etc.*), spray drying is preferred because it is economical, flexible, of easy operation and control, with high drying rates and very short residence times [8]. Therefore, heat sensitive products can be dried at relatively high temperatures while retaining some of their properties such as taste, color, odor and nutrients [9,10]. However, drying of sugar-rich foods products is difficult due to the high water content, low molecular weight and low glass transition temperatures (T_g_) [11]. These products are very sensitive to the processing conditions and may adhere to the walls of the dryer forming an agglomerated powder. This leads to low processing yields and adverse product quality [12,13]. Thus to avoid this type of unwanted conditions such as sticking problems, collapse of the microstructure and damage during storage, the physical and thermal properties of polysaccharides like MXs must be determined first.

Therefore, the goal of this study was to establish a relation between the DP and the physicochemical properties of MXs with different DE. For this purpose, MXs powders were initially chemically characterized for determining the DP and their total number of glucose units. Then, the effect of water adsorption on the physical and thermal properties was determined for the different MXs. It is expected that the results obtained in the study will prove useful to identify the potential applications of MXs according to the DP and to establish optimal storage conditions. These results may be of importance when using MXs as an additive in the drying process, as carrier agents in the encapsulation of proteins, and in the development of food products with high sugar content.

## 2. Results and Discussion

### 2.1. Determination of the Degree of Polymerization of Powdered Maltodextrins

**Figure 1:**
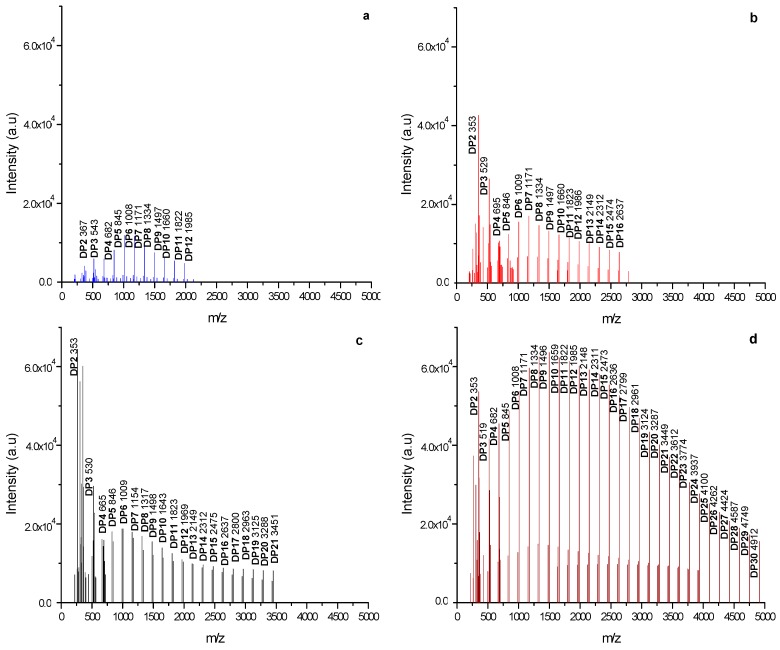
MALDI-TOF spectra of MXs: (**a**) Mc; (**b**) M10; (**c**) M20 and (**d**) M40.

The initial state of MXs powder is of major importance to the final properties and applications. Properties such as water adsorption, crystallinity and glass transition temperature depend in turn on the degree of polymerization (DP). As known, synthetic polymers have a molecular weight distribution (MWD) which can be characterized by the weight-average molecular weight (M_w_), the number-average molecular weight (M_n_) and polydispersity. MALDI-TOF mass spectrometry has shown to be an accurate technique to characterize the MWD of carbohydrate polymers [14]. Moreover, Kazmaier *et al.* [15], studied a set of maltodextrins with a DP in the range of 2–13, and concluded that MALDI-TOF mass spectrometry is best suited technique for the detection of high molecular weight species such as oligosaccharides. Figure 1a–d show the mass to charge ratio (*m*/*z*) spectra obtained from the MALDI-TOF analysis of the MXs powders. On the *x*-axis is plotted the molecular weight, while in the *y*-axis is the number of molecules having the given molecular weight. The spectra show adjacent peaks related to the thermal fragmentation of inulin separated by a mass interval of 162 g/mole. This value is related to the loss of water produced by the hydrolysis of the polysaccharide during the thermal fragmentation (M_w glucose_−M_w water_). As observed in the figures, the spectrum from the M40 sample exhibited a greater degree of fragmentation, which qualitatively indicates larger chains of glucose units with a value up to 4911 (*m*/*z*). Contrary to this, the Mc sample exhibited the shorter fragmentation with a value up to 1985 (*m*/*z*). Average molecular weight values M_w_ and M_n_ were calculated from the MADI-TOF spectra according to the Equations (1) and (2), respectively:

M_w_ = Σ(N*i*M*i*^2^)/Σ(N*i*M*i*) (1)

M_n_ = Σ(N*i*M*i*)/ΣN*i* (2)

where N*_i_* represents the intensity of the *i* peak and M*_i_* is the mass of the oligomer. The polydispersity (X_n_) was calculated from the ratio of the molecular weights (M_w_/M_n_) [16]. The DP value was calculated by dividing the mass of the *i* oligomer by the mass interval (162 g/mole). The M_w_, M_n_, X_n_ and DP were calculated with the aid of the appropriate software (Polytoos v.10) and the values are reported in Table 1. For comparison purposes, the DE values reported by the supplier of the maltodextrins and the DE values determined experimentally in this work are included in Table 1. The experimentally determined values showed good agreement with those reported by the supplier. As expected, the MWD for MXs showed the typical behavior of synthetic polymers, where M_n_ presented lower values than those for M_w_. The calculated M_w_ and M_n_ showed values in the range of 1335–2873 and 1168–2242 g/mole, respectively. The four MXs exhibited close to unitary polydispersity values, which indicates a narrow molecular weight distribution. For samples Mc, M10 and M20, the X_n_ slightly increased with the DE, showing the extent of the hydrolysis of starch. However, for sample M40, the value decreased slightly to 1.28. The DP range was between 12 and 30, and the values increased linearly with the molecular weight. These results are in agreement with those reported by Meuter *et al.* [17], who studied maltodextrins with DP values in the range of 1–7, and the corresponding increase in the M_w_ range of 180–1152 g/mole. When comparing the relation between the DE and DP, our results showed a linear relation. Accordingly to Kennedy *et al.* [18], the chemical composition of maltodextrins is affected by the type and conditions exerted during the hydrolysis. In consequence, MX-based products with similar DE value may present different physical properties [19]. Anyway, it has been shown that the DE is not the best parameter for predicting the performance of maltodextrins [20]. Several methodologies have been employed for setting a relation between the MWD and DE of MXs, showing remarkably variable results. Rong, Sillick and Gregson [4], determined both M_n_ and DE from osmometry measurements of MXs with a DP up to 7, and found an inverse relation between the calculated parameters. Sun *et al.* [2], employed high-performance liquid chromatography (HPLC) and gel permeation chromatography (GPC) for determining MWD of MXs in the DE range of 7–28, finding an uneven variation between the molecular distribution and the composition of oligosaccharides. Recently, Silva *et al.* [21] reported the structural analysis of commercial grade maltodextrins by MALDI-TOF and size exclusion chromatography (SEC). They determined the neutral sugar content (acetylated and alditol acetate) by gas chromatography (GC), identified the corresponding links from the linear chains (1–4 amylose) and the branched amylopectin chains (1–4 Glcp, 1–6 Glcp and 1–4,6 Glcp). With these results, they calculated the percentage of branching of the main chain and established that the higher the branching the higher the hydrodynamic volume (OH groups) and in consequence the higher molecular weight. During the hydrolysis of starch, both processes may occur simultaneously, the breaking of the polymer chain and the subsequent polymerization of the chains into amylose and amylopectin. Depending on the arrangement of these chains the resulting maltodextrin may be linear or branched. Thus, a high DE value indicates more available functional groups and a higher percentage of branching. Our results showed a linear increase in the DP as the DE was increased. This finding suggested that branching increased for the MXs samples in the following order Mc, M10, M20 and M40.

Certainly, the characterization by mass spectroscopy yielded important data about the chemical structure of the different MXs, determining the number of glucose oligomers contained in the carbohydrate polymer chains. From the technological point of view, this is essential in order to establish the potential applications for commercial grade MXs. Additionally, these results were of importance to understand that there is not a direct relation between the DP and DE, and from these parameters, the DP provided a better insight about the chemical structure of the MX.

**Table 1:** Molecular weight parameters from the MXs, determined from MALDI-TOF spectrometry experiments.

Maltodextrin	DE ^1^ (%)	DE ^2^ (%)	MALDI-TOF			
			M_w_ (g/mole)	M_n_ (g/mole)	X_n_	DP (Units of Glucose)
Mc	------	7 ± 1.02	1335 ± 106	1168 ± 93	1.14	2–12
M10	9–14	10 ± 1.98	1625 ± 113	1205 ± 84	1.34	2–16
M20	18–22	21 ± 2.04	1925 ± 96	1324 ± 66	1.45	2–21
M40	37–42	39 ± 1.27	2873 ± 172	2242 ± 134	1.28	2–30

Dextrose Equivalent (DE): ^1^ Values reported by the commercial supplier; ^2^ Values determined experimentally as described in Section 2.1.

### 2.2. Sorption Isotherms

**Figure 2:**
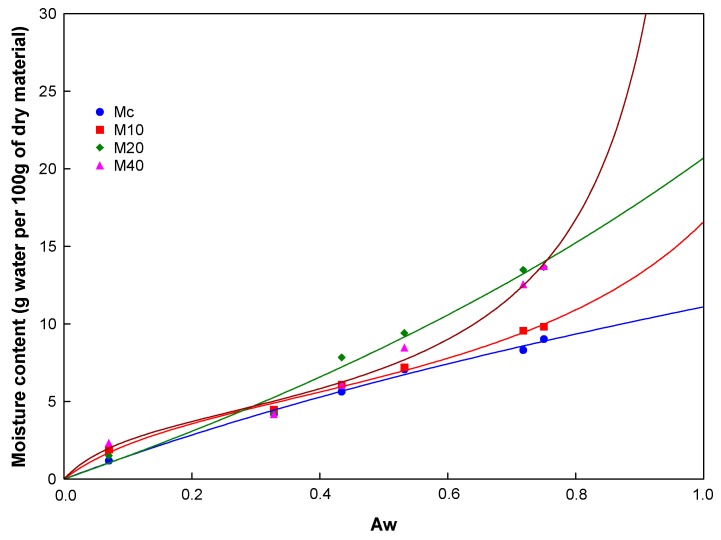
Moisture adsorption isotherms at 30 °C for MXs with different DP. The continuous line indicates the regression according to the GAB model.

Figure 2 shows the moisture content at equilibrium for water activities (a_w_) from 0.070 to 0.75, for the MXs (Mc, M10, M20 and M40) at 30 °C. The equilibrium water content (a_w_) is expressed as the water absorbed (in grams) per 100 g of dry sample. Mainly two types of models are employed to describe this type of isotherm: the BET and the GAB [22] equations. The BET equation better reproduces the experimental behavior at a_w_ in the range of 0.05 < a_w_ < 0.40, but hinders the fitting of the experimental data over the whole range of water activities. Unlike the BET model, the GAB equation includes the experimental data of a_w_ up to a value of 0.9. Thus, in this study the GAB model was used for fitting the adsorptions isotherms according to Equation (3):

*m*/*m*_0_ = *ck*a_w_/[(1 − *k*a_w_)(1 – *k*a_w_ + *ck*a_w_)] (3)

In Figure 2, the continuous line connecting the scattered data points represents the regression according to the GAB model. On the other hand, Brunauer, Deming, Deming and Teller [23], established five types of isotherms based on the adsorption of Van der Waals gases: Type 1 corresponds to chemisorption phenomena that take place in a single layer at the surface-active sites. Type II and III are frequently associated for nonporous food powders, while type IV and V for porous products. Mc sample exhibited an adsorption behavior corresponding to curve Type I. The amount of water absorbed was from 1.6 to 8.2 g of water per 100 g of dry sample at a_w_ in the range of 0.07–0.75. Likewise, samples M10 and M40 exhibited a Type II isotherm known as sigmoid, which is characteristic of soluble products that show an asymptotic trend as the water activity approaches to the unit. These samples (M10 and M40) showed equilibrium water contents of 1.89–9.81 and 2.33–13.72 g of water per 100 g of dry sample, respectively. Sample M20 exhibited an equilibrium moisture content of 1.50–13.66 g of water per 100 g of dry sample, which corresponded to the Type III isotherm in the interval previously stated for a_w_. According to Fabra *et al.* [24], this behavior is typical of sugar-rich products, which adsorb small amounts of water at low water activities, but exhibit a steady increase in the amount of water adsorbed at higher values of a_w_. Similar isotherms for single maltodextrin systems and mixtures with other components such as juice, proteins and lipids, have been reported in several works [1,25,26].

Three parameters (*m*_0_, *c* and *k*) characterize the interaction of water with the constituents of the food: *m*_0_ is the water content at the monolayer level, *c* is characteristic of the product and it is related to the adsorption heat of the monolayer, and *k* is a correction factor related with the desorption heat of the monolayer [26]. Table 2 shows the values of *c*, *k*, *m*_0_ and the correlation coefficient (r^2^). Accordingly to Lewicki [27], if these values are in the range of 5.67 ≤ *c* ≤ ∞ and 0.24 ≤ *k* ≤ 1, then the predicted value for the monolayer will vary only ±15% of its actual value. Thus, after comparing the values of the *c* and *k* constants for the systems analyzed in this work, it was clearly observed that the constant values for samples M10 and M40 were between the limits established by Lewicki. This indicated a good prediction of the monolayer water content from the adsorption isotherms. However, for samples Mc and M20 the values of the constants deviated from that established by Lewicki. These observations corroborated that the values of *m*_0_ predicted by the GAB model were reliable only for the M10 and M40 samples, which showed values very close to 5 g of water per 100 g of dry powder. Additionally, the sigmoidal curve may be divided into three zones, each one representing an adsorption phenomenon. In zone I, corresponding to water activity in the range of 0–0.25, water monolayer is strongly adsorbed on the surface of the solid by hydrogen bond interactions. This water monolayer is difficult to eliminate by dehydration or to immobilize by freezing. The boundary between zone I and II is associated to the monolayer water level. For zone II in the range of a_w_ of 0.25–0.8, more water molecules are added forming multilayers, observing capillary condensation on the surface of the solid. Zone III is presented at a_w_ values greater than 0.8; the proportion of water is such that the liquid phase is observed. As observed in Figure 2, the equilibrium monolayer content determined graphically at a_w_ of 0.25 is very similar for all the MXs analyzed in this work, with a water content about 4–5 g of water per 100 g of dry sample. These results indicated that the MXs analyzed herein showed a similar adsorption behavior at low a_w_, but behaved differently at a_w_ higher than 0.43. Certainly, water adsorption in sugar rich products is largely influenced by the chemical structure and the availability of the active sites on the surface of the powder particles [28].

**Table 2:** Fitting constants of the GAB model for water adsorption in the MXs.

Maltodextrin	GAB Model Parameters			
	*c*	*k*	*m*_0_	r^2^
Mc	6.26634	0.10599	23.29	0.997
M10	8.647117	0.698417	5.25	0.996
M20	1.098873	0.31986	41.33	0.992
M40	11.58617	0.94973	4.12	0.993

Martínez-Navarrete *et al.* [29] concluded that this type of curves can be useful for determining the rate of deterioration in food products such as milk powder and fruits (kiwi and strawberry), and found that the product is more stable at a_w_ of 0.15–0.25. Saavedra-Leos *et al.* [12] established that adsorption isotherms together with the results from other characterization techniques such as thermogravimetric analysis (TGA), modulated differential scanning calorimetry (MDSC), X-ray diffraction (XRD), scanning electron microscopy (SEM) and optical microscopy (OP) were useful in the developing of new food products and the prediction of product stability. Moreover, with these isotherms it was possible to determine the maximum water content at which the physical properties of the powder product may remain unchanged during storage.

### 2.3. Simultaneous Thermal Analysis (TGA-DSC)

**Figure 3:**
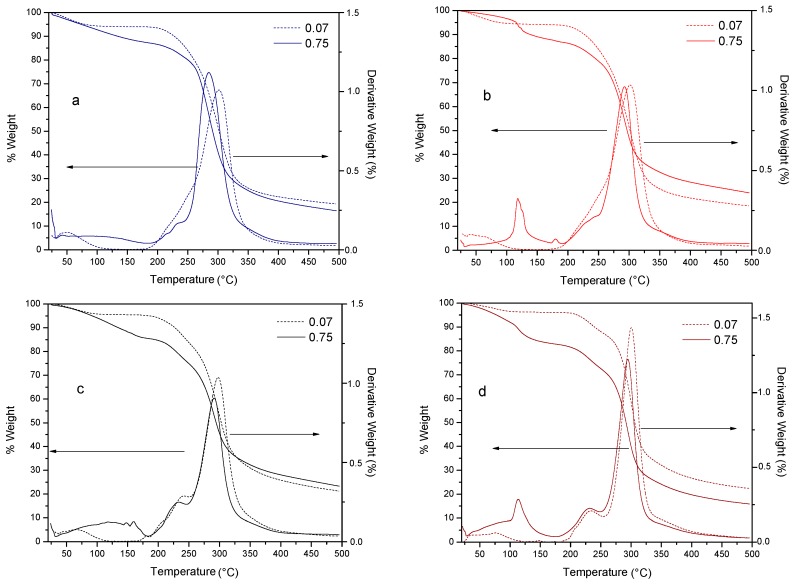
Thermal decomposition of MXs. TGA and DTG curves for: (**a**) Mc; (**b**) M10; (**c**) M20, and (**d**) M40.

With the aid of the thermogravimetric analysis (TGA) along with its derivative (DTG) it is possible to determine the thermal degradation temperature T_d_ of the different species contained in a sample. Particularly, the TGA analysis allows the determination of mass losses that are related to the thermal stability, being the decomposition a thermal phenomenon that starts at T_d onset_. The DTG allows separating thermal events occurring in a narrow temperature range. Figure 3 shows the TGA and DTG of the MXs studied in this work, corresponding to a_w_ of 0.07 and 0.75.

In the TGA curve, the first thermal event observed was in the temperature range of 70–110 °C, associated with a first order transition such as evaporation and corresponding to approximately 3.5%–7% of water loss. A second and more pronounced thermal event was presented in the temperature range of 200–350 °C, associated with a mass loss of about 65%–70%. This mass loss was attributed to the thermal decomposition (T_d_) of long molecular chains, the polymerization processes and the isomerization reactions associated with dehydration [30]. In Table 3 are summarized the decomposition temperatures for the MXs at the extremes a_w_ values (0.07 and 0.75). At low water activity, the onset (T_d Onset_) of the decomposition occurred in the range of 163–180 °C, whereas the peak (T_d Peak_) was observed about 300 °C. Although it is well known that the thermal decomposition mainly depends on the homogeneity in the chemical composition and the MWD of the carbohydrate polymer, at higher water activity some differences in both T_d Onset_ and T_d Peak_ were observed. These variations were attributed to the thermal hydrolysis induced by the large amount of adsorbed water and the supplied heat. The hydrolysis was observed as a broad band or as a well-defined peak at a temperature about 115 °C, and was presented only at a_w_ of 0.75. The last feature observed in the DTG curve, it was a small decomposition peak presented about 230–240 °C. This event was identified as the thermal decomposition of the low molecular weight oligosaccharides and clearly it is related with the MWD, since samples M10, M20 and M40 showed a high content of oligosaccharides with a DP of 2. At the higher water activity, this peak was observed for all the samples studied, confirming the aforementioned thermal hydrolysis.

**Table 3:** Thermal decomposition temperatures of MXs at the extreme water activities (a_w_), determined from TGA-DTG curves in Figure 3.

Maltodextrin	a_w_	T_d onset_ (°C)	T_d peak_ (°C)
Mc	0.07	180.1	301.4
	0.75	190.1	284.7
M10	0.07	165.2	301.7
	0.75	192.8	292.4
M20	0.07	163.4	297.6
	0.75	182.1	291.2
M40	0.07	180.9	300.4
	0.75	181.8	294.6

These results were of importance to set the decomposition temperature range at which the MXs can be heated for avoiding chemical changes in the product. Additionally, other thermal events were identified and related to the decomposition phenomena occurred during the heating and caused by the adsorption of water.

### 2.4. Modulated Differential Scanning Calorimetry (MDSC)

**Figure 4:**
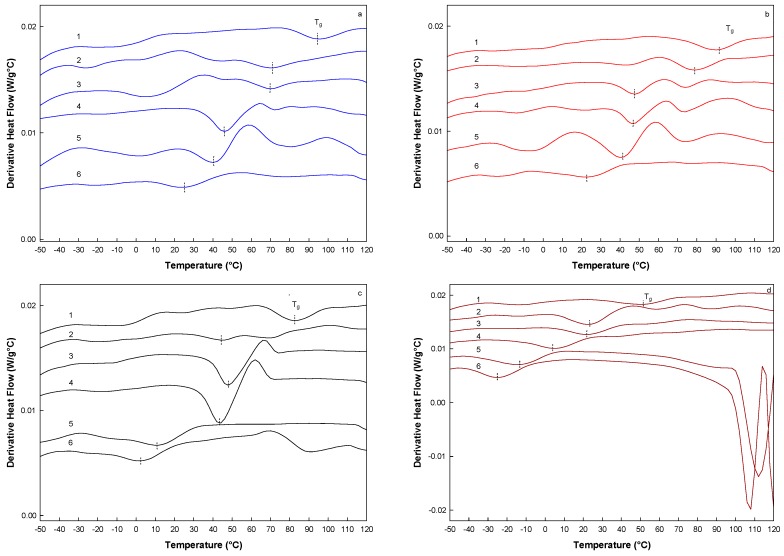
Thermograms of the first derivative of the MDSC for the MXs at the different a_w_. (**a**) Mc; (**b**) M10; (**c**) M20; and (**d**) M40. Water activities are indicated by the numbers: (1) 0.070, (2) 0.328, (3) 0.434, (4) 0.532, (5) 0.718, and (6) 0.75. The small dotted vertical line on each curve indicates the temperature at which T_g_ was determined.

The glass transition temperature (T_g_) was determined by modulated differential scanning calorimetry (MDSC), which is an extension of the conventional DSC, and it has been reported as a suitable technique for thermal characterization of biopolymers with complex thermal events [31,32]. Thus, due to the sinusoidal modulation of the temperature, MDSC is widely employed in detecting T_g_. Figure 4 shows the MDSC thermograms of the MXs, which was determined by calculating the first derivative of the heat flow with respect to temperature. It was clearly observed that for a given MX, T_g_ decreased as a_w_ increased. For each of the MXs the T_g_ decreased as 100–20 °C, 90–20 °C, 80–0 °C and 52–(−20) °C, for Mc, M10, M20 and M40, respectively. This is a normal behavior observed in sugar-rich systems such as inulin, attributed to the plasticizer effect of water promoted by the formation of hydrogen bonds on the hydrophilic groups [12]. This phenomenon causes an increase in the intermolecular free volume space, diminishing the viscosity of the system and increasing the molecular mobility [33]. Other feature observed was the decrease in the T_g_ with the DE. This trend was also observed by other authors [5,34]. Busin *et al.* [35] based on theoretical calculations proposed a linear relationship between the T_g_ and DE for maltodextrins within the DE range of 2–100. Our results indicated a deviation of about 68 °C below those values calculated by Busin *et al.* [35]. However, the MWD is a more appropriate parameter for predicting the T_g_, which is associated with the mobility of the chains [36]. The results reported herein showed a linear decreasing tendency between both M_w_ and M_n_, and the corresponding T_g_ value. Similar results were reported by Meuter *et al.* [17], for the T_g_ of the dextrose-maltodextrin system at different DP in the range of 1–7 and corresponding M_w_ of 180–1152 g/mole. They found that the highest T_g_ value was registered for the maltodextrin with DP of 5 and not for that with a DP of 7. 

In addition, these results suggested a similar behavior observed in the glucose isomers such as sucrose, maltose and lactose, which present the same molecular weight but due to differences in the chemical structures, exhibit very different physical properties. Likewise, polysaccharides such as inulin (based on fructose chains) and maltodextrins (based on glucose chains) showed an opposite behavior between the MWD and the T_g_. This difference may be caused by the way in which the polymer chains are arranged at the microstructure level and the position of the functional groups available to establish *inter* and *intra*-molecular interactions. The technological application of these results lies in the possibility to establish the use of a MX according to the MWD and physical properties such as the T_g_. Thus, for a process where it is needed to keep the microstructural stability at temperatures below 90 °C, the low molecular weight MXs (Mc and M10) might be appropriate. An example of this, it is the spray drying process, where the structural rigidity is desired for avoiding particle agglomeration and sticking on the walls of the dryer. In the other hand, the high molecular weight MXs (M20 and M40) may be employed as additive in low temperature processes such as volume enhancers in confectionery and viscosity promoters in syrups.

### 2.5. Microstructural Analysis

**Figure 5:**
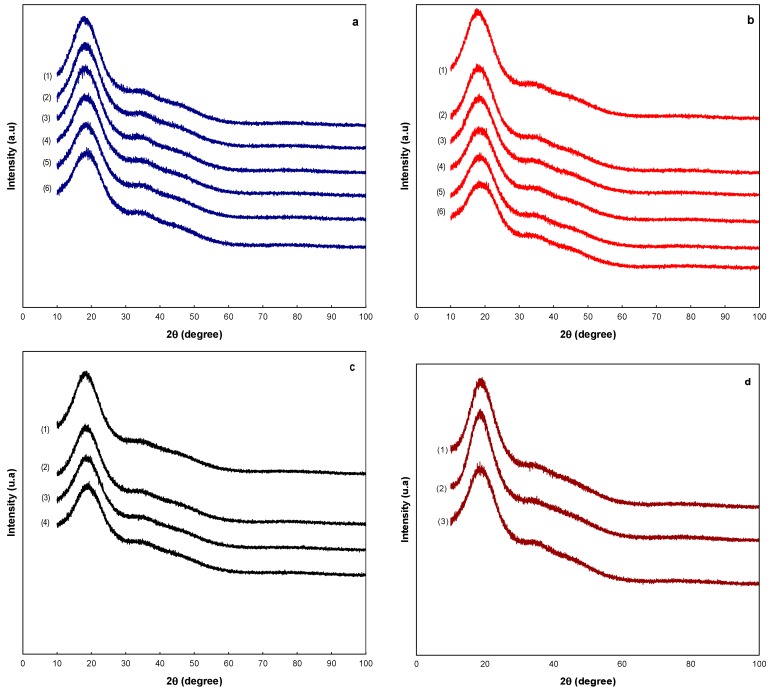
Analysis of the microstructure of the MXs by XRD: (**a**) Mc; (**b**) M10; (**c**) M20; and (**d**) M40. Water activities are indicated by the numbers: (1) 0.070, (2) 0.328, (3) 0.434, (4) 0.532, (5) 0.718, and (6) 0.75.

One of the techniques used to elucidate the state of the microstructure is by X-ray diffraction. Figure 5 shows the diffraction patterns for the MXs at the different a_w_. The Figure 5 includes only the diffraction patterns of the MXs samples that remained in the solid state after the adsorption of water at the given a_w_, *i.e.*, only those samples where saturation of water was not presented. It was observed that all the diffraction patterns showed a broad peak about 2θ of 15° and the absence of well-defined peaks.

**Figure 6:**
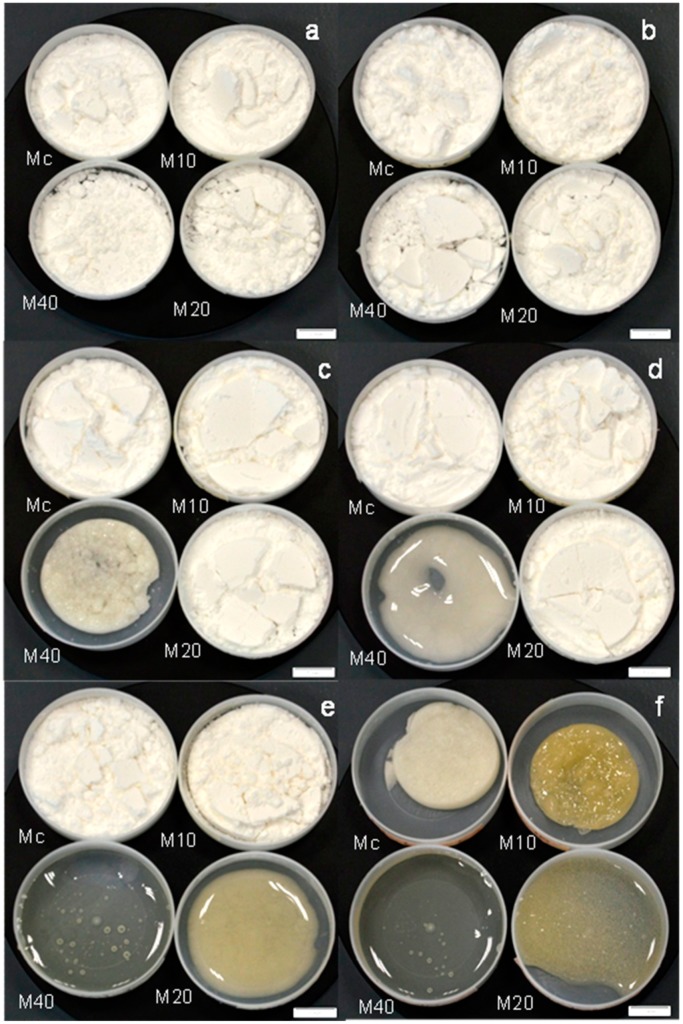
Optical micrographs of the MXs at the different water activities: Water activities are indicated by the letters: (**a**) 0.070; (**b**) 0.328; (**c**) 0.434; (**d**) 0.532; (**e**) 0.718; and (**f**) 0.75. The white scale bar is equal to 2 cm.

These observations indicated the conservation of the amorphous microstructure in the samples at all the water activities. This is a very important finding because unlike inulin, which is composed of fructose chains and crystallizes with the adsorption of water [12], the MXs which are composed of glucose chains remained amorphous despite the amount of adsorbed water. The aforementioned changes in the microstructure were observed macroscopically as the evolution of the powder’s appearance. Figure 6 shows images of the MXs powders at the different water activities acquired with an optical microscope. Initially, the MXs were observed as well-dispersed powders *i.e.*, non-agglomerated, and with a white color appearance. For the maltodextrins Mc, M10 and M20, this appearance remained unchanged up to a water activity about 0.532; above this value the overall appearance began to change. At a_w_ of 0.718, the Mc sample was still observed as a dispersed powder but with a yellowish color, while at 0.75 the powder totally changed its appearance, observing the overall volume contraction and a gray color. For the M10 sample these changes were more notorious, particularly the change of color. 

The M20 sample presented the yellowish color at a_w_ of 0.532, and then at a_w_ of 0.718 the volume contraction was accompanied by the saturation of the powder with liquid water. The further increase of humidity at a_w_ of 0.75 showed the dissolving of the solid and the presence of the liquid state. The M40 MX showed all these changes at earlier stages of water adsorption. At a_w_ of 0.434 was observed the volume contraction and at a_w_ of 0.532, the powder was fully saturated of water. Above this water activity, the M40 powder was dissolved, and the liquid phase was observed. According to the X-ray diffraction (XRD) and optical images results, the MXs did not show any changes in their microstructure *i.e.*, crystallization, but just the transition from the solid state into a rubbery-like state. In this state, there is a high molecular movement and the powder samples may present several changes such as agglomeration, loss of volatiles, color changes, stickiness and accelerated chemical reactions. The rubbery state is seen at temperatures above the glass transition. The results from the MDSC measurements showed good agreement with the microstructural analysis results. Clearly, the adsorption of water decreased the T_g_ of the MXs systems, modifying the amorphous microstructure from the glass into the rubbery state, where the different changes in appearance were observed. All of these observations in the physical states could be correlated with the MWD of the MXs. For example, the low molecular weight MXs (Mc and M10) showed a lower adsorption of water and in consequence the conservation of the microstructure at relative high water activities. In the other hand, higher molecular weight MXs which presented more available sites for water adsorption, showed larger adsorptions of water. As a result, the changes in the physical state were observed at low water activities.

These results have provided great information about the changes in the physical state of the MXs, which may be helpful for selecting a maltodextrin for a particular technological application, based on the ease of being able to visually relate the evolution of the microstructure with the adsorption of water.

## 3. Experimental Section

### 3.1. Sample Preparation

Four maltodextrin powders extracted from corn starch and with different DE were employed in this work. The powders were identified as commercial maltodextrin (Mc), and according to the dextrose equivalent: DE 10 (M10), DE 20 (M20) and DE 40 (M40). Mc powder was acquired from INAMALT (Gualadajara, Mexico) and M10, M20 and M40 from INGREDION Mexico (Guadalajara, Mexico). DE was determined by triplicate according to a standard method based on titration with Fehling’s solution and methylene blue as indicator [37].

### 3.2. Mass Spectrometry Analysis

Molecular mass and molecular mass distributions were determined by mass spectrometry (MS) analyses in a high performance Matrix-assisted Laser desorption/ionization (MALDI) system equipped with a time-of-flight (TOF) mass spectrometer (Autoflex Speed, Bruker Corp., Billerica, MA, USA). MX powder (1 mg) was dissolved in chloroform (1 mL) and mixed with 1.5 μL of saturated solution of 2,5-dihydroxybenzoic acid. The solution was deposited onto the MALDI plate and irradiated with a pulsed nitrogen laser (337 nm) for desorption and ionization. 500 laser shots were averaged for each sample to obtain the representative mass to charge (*m*/*z*) spectra. Molecular weight distributions were determined from MALDI-TOF using the Polytoos v.10 software (Bruker). Each analysis was repeated three times.

### 3.3. Sorption Isotherms

From each dried sample, approximately 2 g MX powder were placed into a closed beaker containing different saturated reagents. The reagents employed for reaching the desired water activity (a_w_) at equilibrium were: NaOH (0.070), MgCl_2_ (0.328), K_2_CO_3_ (0.434), Mg (NO_3_)_2_ (0.528), SrCl (0.718) and NaCl (0.75). Incubation temperature was chosen based on an average storage temperature, considering the range of 25–35 °C as the room temperature commonly used in warehouses. Dried samples were left for incubation for 30 days at 30 °C [38]. After the incubation time was elapsed, a_w_ was determined into an Aqualab Series 3 Water Activity Meter (Decagon Devices, Inc., Pullman, WA, USA). Water content was measured accordingly to the AOAC method, which requires drying the sample at an oven at 110 °C for 2 h. Each experiment was performed in triplicate.

### 3.4. Thermal Analysis

Thermogravimetric (TGA) and Differential Scanning Calorimetry (DSC) analyses were carried out in a simultaneous TGA-DSC SDT Q600 (TA Instruments, New Castle, DE USA). For DSC, baseline was calibrated with Indium (melting temperature of 156.6 °C and melting enthalpy of 28.47 J/g). Samples of 5–10 mg were encapsulated in standard aluminum pans. Thermograms were recorded at a heating rate of 5 °C/min over a range of 25–500 °C. Using the Universal Analysis 2000^©^ software, different features from the curves were identified: mass loss (% *w*/*w*), initial and final peak melting temperatures (T_m onset_, T_m peak_ and T_m final_, respectively), and initial and final peak degradation temperatures (T_d onset_, T_d peak_ and T_d final_, respectively).

### 3.5. Modulated Differential Scanning Calorimetry (MDSC)

A modulated DSC Q200 (TA Instruments, New Castle, DE, USA) equipped with an RCS90 cooling system was employed for accurately determining the glass transition temperature (T_g_) of MXs samples. The instrument was also calibrated with Indium for melting temperature and enthalpy, meanwhile Sapphire was used as the standard for the heat capacity (C_p_) calibration. Samples ranging in 5–10 mg were encapsulated in Tzero^®^ aluminum pans. Thermograms were acquired at a temperature range of −90 to 250 °C, with a modulation period of 40 s and temperature amplitude of 1.5 °C. Each experiment was repeated three times.

### 3.6. Structural Analysis by X-Ray Diffraction (XRD)

The structure of maltodextrin samples was qualitatively determined by XRD analysis in an X’Pert Empyrean diffractometer (PANalytical, Almelo, The Netherlands) with Cu-Kα radiation (*l* = 1.5406 Å) operated at 45 kV, 40 mA and equipped with a X’Celerator detector in a Bragg-Brentano geometry. Scans were performed in the 2θ range of 10°–100°, with step size of 0.016° and 20 s per step.

### 3.7. Optical Microscopy

The overall morphology and appearance were evaluated by optical microscopy with a stereomicroscope SZX16 (Olympus^©^, Melville, NY, USA). Micrographs were acquired at 2X with a digital camera of 12 Mpx.

## 4. Conclusions

Four maltodextrin (MX) systems with different dextrose equivalents (DE) and degrees of polymerization (DP) were studied in this work. The technological application for each maltodextrin was provided based on the characterization of the physical properties. The results showed a direct relation between the DE and the DP. Of these two parameters, the DP provided a better understanding of the microstructure of the MXs. The adsorption performance of the MXs was similar at low water activities (a_w_), but varied very much at higher a_w_. These variations were attributed to the differences in the DP, where the larger DP presented the more available sites for water adsorption. The decomposition temperature (T_d_) was determined from the thermogravimetric analysis (TGA), finding that the combined effect of water adsorption and heat, induced the thermal hydrolysis of the low molecular weight species contained in the MXs. The glass transition temperature (T_g_) was accurately determined by modulated differential scanning calorimetry (MDSC), observing a linear decreasing tendency between the molecular weights and the corresponding T_g_. This behavior suggested differences in the way the polymer chains of glucose units were arranged at the microstructural level. The microstructural analysis by XRD showed the conservation of the amorphous structure at all a_w_. These results indicated that the glucose units in the DP range of 2–30 did not crystallize with the adsorption of water, which is an abnormal behavior observed in other sugar-rich systems. The evolution of the amorphous glassy structure into a rubbery state was macroscopically monitored by optical microscopy, clearly observing changes in the overall appearance of the MXs powders. All of these results allowed to set the potential applications for each MX based on the DP.
